# Antisepsis and Antiseptics

**Published:** 1895-04

**Authors:** 


*Antisepsis and Antiseptics.* Buchanan, Terhune Co., Newark, N. J. The busy practitioner of veterinary science will find this concise, well-filled little book well worth reading. Besides brushing one up on rusty points and conveying much valuable knowledge, it epitomizes the history of antiseptic medicine in a brief, interesting, and concise manner. The future of surgery under the better searchlight of antisepsis will continue to prove a daily record of valuable accessions and triumphs in modern surgery.

